# Liquid–liquid phase separation of full-length prion protein initiates conformational conversion *in vitro*

**DOI:** 10.1016/j.jbc.2021.100367

**Published:** 2021-02-02

**Authors:** Hiroya Tange, Daisuke Ishibashi, Takehiro Nakagaki, Yuzuru Taguchi, Yuji O. Kamatari, Hiroki Ozawa, Noriyuki Nishida

**Affiliations:** 1 Department of Molecular Microbiology and Immunology, Graduate School of Biomedical Sciences, Nagasaki University, Nagasaki, Japan; 2 Department of Neuropsychiatry, Graduate School of Biomedical Sciences, Nagasaki University, Nagasaki, Japan; 3 Life Science Research Center, Gifu University, Gifu, Japan

**Keywords:** prion, liquid–liquid phase separation, liquid–solid phase transition, amyloid, aqueous two-phase system, ATPS, aqueous two-phase system, BH, brain homogenate, DIC, differential interference contrast, IDR, intrinsically disordered regions, LLPS, liquid–liquid phase separation, PEG, polyethylene glycol, PK, proteinase K, PrP^C^, normal prion protein, PrP-res, protease K-resistance prion protein fragment, PrP^S^^c^, misfolded prion protein, rPrP, recombinant prion protein

## Abstract

Prion diseases are characterized by the accumulation of amyloid fibrils. The causative agent is an infectious amyloid that comprises solely misfolded prion protein (PrP^Sc^). Prions can convert normal cellular prion protein (PrP^C^) to protease K-resistance prion protein fragment (PrP-res) *in vitro*; however, the intermediate steps involved in this spontaneous conversion still remain unknown. We investigated whether recombinant prion protein (rPrP) can directly convert into PrP-res *via* liquid–liquid phase separation (LLPS) in the absence of PrP^Sc^. We found that rPrP underwent LLPS at the interface of the aqueous two-phase system of polyethylene glycol and dextran, whereas single-phase conditions were not inducible. Fluorescence recovery assay after photobleaching revealed that the liquid–solid phase transition occurred within a short time. The aged rPrP-gel acquired a proteinase-resistant amyloid accompanied by β-sheet conversion, as confirmed by Western blotting, Fourier transform infrared spectroscopy, and Congo red staining. The reactions required both the N-terminal region of rPrP (amino acids 23–89) and kosmotropic salts, suggesting that the kosmotropic anions may interact with the N-terminal region of rPrP to promote LLPS. Thus, structural conversion *via* LLPS and liquid–solid phase transition could be the intermediate steps in the conversion of prions.

Transmissible spongiform encephalopathies, also called prion diseases such as Creutzfeldt–Jakob disease (CJD) in humans, are infectious and fatal neurodegenerative diseases with rapidly progressive dementia (1). Transmissible spongiform encephalopathies are characterized by the accumulation of misfolded prion protein (PrP^Sc^), which is spontaneously converted from normal prion protein (PrP^C^). PrP^C^ is well preserved among mammalian species and is particularly expressed in neurons and tethered to the cell membrane *via* the glycosylphosphatidylinositol anchor (2). The protein-only hypothesis proposes that the infectious agent prion is solely composed of PrP^Sc^. The main biochemical characteristics of PrP^Sc^ are that it has seeding activity to convert PrP^C^ into itself (PrP^Sc^) and rPrP into protease K-resistance prion protein fragment (PrP-res); both of them have protease K resistance. However, PrP-res does not necessarily correlate with infectivity or seeding activity (3, 4, 5). This conversion process presumably proceeds *via* direct interaction between PrP^C^/rPrP and PrP^Sc^ (6). Several studies have attempted to generate artificial PrP^Sc^, and the amplification of PrP^Sc^ *in vitro* has been successfully demonstrated using intermittent ultrasonication on brain homogenates (BHs), called protein misfolding cyclic amplification (PMCA) (7, 8). Not only sonication but also shaking of the protein solution can promote *in vitro* amyloid formation. The quaking-induced conversion (QuIC) assay is now widely used to detect trace amounts of PrP^Sc^ in cerebrospinal fluid using rPrP as a substrate (9). These lines of experimental evidence suggest that rPrP can be converted to proteinase K (PK)-resistant amyloid (rPrP-res) in the presence of PrP^Sc^, with the provision of kinetic energy. However, to explain spontaneous generation and to generate artificial prions, the spontaneous misfolding process from rPrP to rPrP-res in the absence of PrP^Sc^ needs to be elucidated.

Recently, proteins with intrinsically disordered regions (IDRs) have been shown to undergo liquid phase separation in the cytoplasm and form membraneless organelles such as stress granules (10). The N terminus of PrP^C^ is an IDR comprising five repeats of proline/glycine-rich sequences, which are called octapeptide repeats. In the liquid phase, IDRs assemble to form a cross-β-sheet structure. The aberrant phase transition of amyloidogenic proteins may facilitate pathological amyloid synthesis. This phenomenon has been associated with the development of neurodegenerative diseases, including Tau protein in Alzheimer's disease and RNA binding protein FUS in amyotrophic lateral sclerosis, which are caused by pathogenic amyloids (11, 12).

In order to elucidate the spontaneous process involved in the conversion of PrP^C^ into PrP^Sc^, we examined whether rPrP can convert into rPrP-res or PrP^Sc^ *via* LLPS, without the use of kinetic energy. In this study, we found that the N-terminal region and kosmotropic anions play an important role in LLPS and liquid–solid phase transition of rPrP. Furthermore, rPrP in gels acquired the features of PrP-res with β-sheet–rich structure and protease K resistance. These results suggest that the LLPS and liquid–solid phase transition can initiate spontaneous conformational conversion of rPrP to PrP-res without the use of kinetic energy.

## Results

### rPrP undergoes liquid–liquid phase separation in the aqueous two-phase system

**Figure 1:**
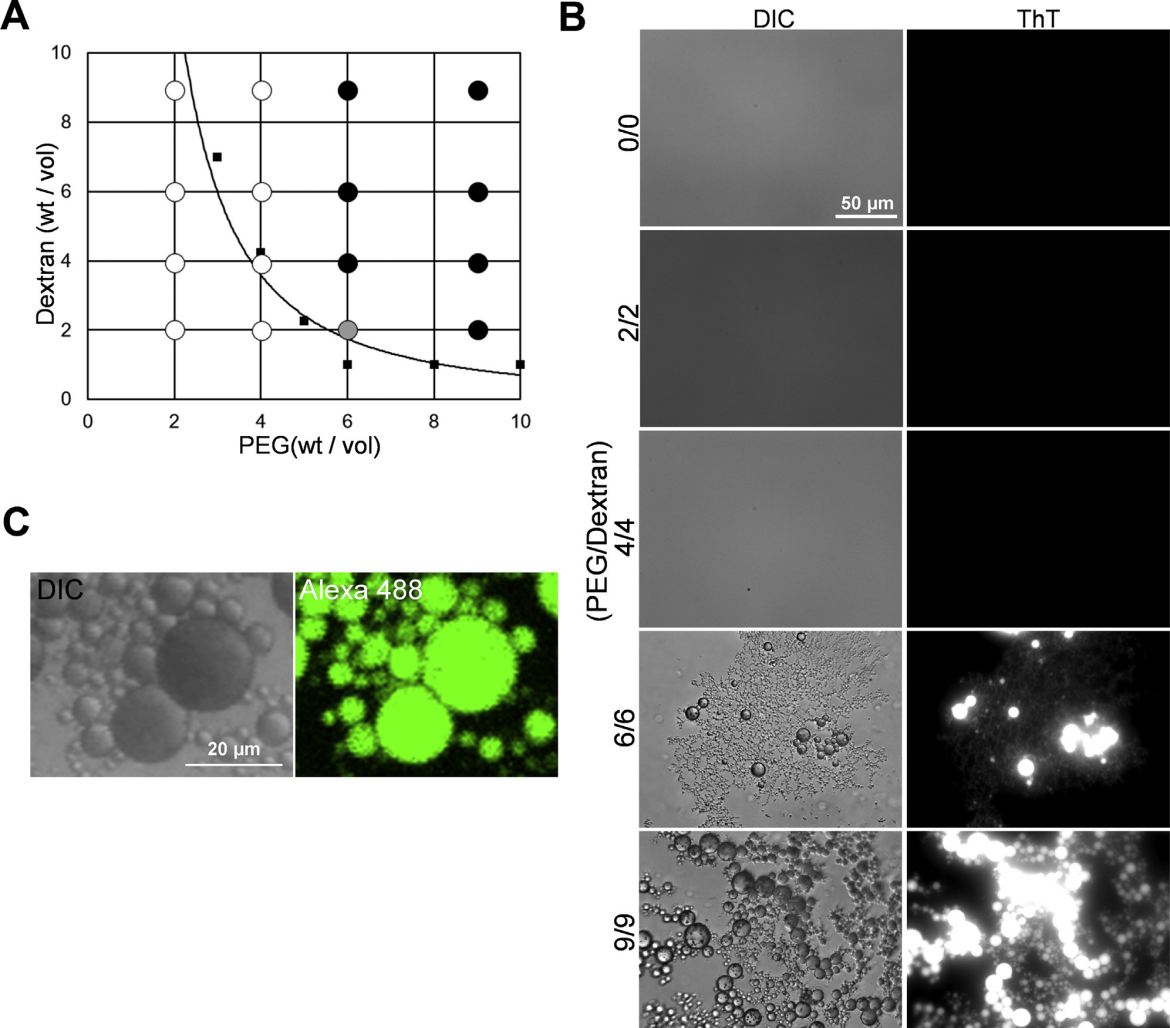
rPrP undergoes liquid–liquid phase separation in an aqueous two-phase system. *A*, phase diagram of an aqueous two-phase system (PEG/dextran). The binodal curve (*solid line*) was drawn with approximation (R^2^ = 0.8626). *Black dots*: rPrP fully underwent liquid phase separation. *Gray dot*: rPrP partially underwent liquid–liquid phase separation with aggregation. *White dot*: rPrP fully aggregated. *Square dots*: average of clouding point. N = 3. *B*, differential interference contrast (DIC) and fluorescence microscopic images of droplets in the interface of PEG/dextran after 24 h of incubation. The scale bar represents 50 μm. *C*, confocal microscopic images of rPrP droplets with Alexa 488–labeled rPrP (1:18). The scale bar represents 20 μm. rPrP, recombinant prion protein.

In general, polymers such as PEG or dextran are used to induce LLPS of proteins as crowding agents (13). First, we tried with a single polymer solution; however, rPrP did not undergo LLPS but resulted in salting out with both PEG and dextran at concentrations greater than 10% (Fig. S1*A*). Next, we applied aqueous two-phase system (ATPS), which is composed of PEG and dextran because we expected that the stronger volume exclusion effect between two different polymers would induce LLPS of rPrP (14). The droplets appeared at the interface of the polymer fractions immediately after mixing 10 μM rPrP with an ATPS mixture containing sodium thiosulfate (Na_2_S_2_O_3_). We tested combinations of various concentrations of the polymers and investigated where ATPS was able to form an interface (15, 16) (Fig. 1, *A* and *B*). Below the binodal curve, no droplet was formed at the interface of the ATPS. Under such conditions (PEG/dextran: 2%–4%/2%–4%), rPrP precipitated as amorphous aggregates at the bottom of wells after 24 h of incubation (Fig. S1*B*). With 6%/6% PEG/dextran, spherical droplets were observed at the interface of ATPS and the bottom of the well, some of them were puddle-like and slightly Thioflavin T (ThT) positive, and the amorphous aggregates were also visualized by ThT. These fresh droplets were visualized by ThT immediately after formation, suggesting that β-sheet formation of rPrP was initiated. Of note, the PEG or dextran droplets in ATPS did not stain with ThT. The ThT-positive droplets appeared even more efficiently with 9%/9% PEG/dextran (Figs. 1*B*, S1*B*). Quantification of ThT fluorescence intensity showed that 9%/9% of PEG/dextran had the highest fluorescence intensity after 24 h of incubation (Fig. S1*C*). Therefore, we set the experimental conditions of 9%/9% PEG/dextran with 120 mM Na_2_S_2_O_3_ in the experiments that followed, unless mentioned otherwise. The ThT-positive aggregates appeared to correlate with the concentration of rPrP for up to 6 μM; spherical droplets with clear ThT fluorescence appeared from 8 μM rPrP and were most prominent at 10 μM of rPrP (Fig. S2*A*). The fluorescence intensity was significantly higher in the presence of 10 μM rPrP (Fig. S2*B*). To confirm if the droplets consisted of rPrP, we performed a similar experiment with Alexa 488–labeled rPrP and found that the fluorescence was equally distributed in all the droplets (Fig. 1*C*).

### Kosmotropic anion species induce droplet formation

We investigated the influence of the salt type on droplet formation and screened various salts according to the Hofmeister series. Sodium salts, such as NaCl, Na_2_S_2_O_3_, Na_2_CO_3_, Na_3_C_6_H_5_O_7_, Na_2_SO_4_, and (NH_4_)_2_SO_4_, were tested. We found that all the salts induced ThT-positive droplet formation, except NaCl and Na_2_CO_3_ (Fig. S3, *A* and *B*). This result suggests that kosmotropic anions were able to induce LLPS of rPrP, whereas Cl^-^ and CO_3_^2-^ did not. Quantification of the fluorescence intensity showed that Na_2_S_2_O_3_ induced significantly higher fluorescence intensity. Because the fluorescence intensity directly reflects the amount of ThT-positive droplets exhibited by ThT, the strength of the fluorescence intensity indicates the efficiency of the droplet formation, suggesting that Na_2_S_2_O_3_ had the best efficiency for droplet formation of rPrP among the salts tested here. Of note, the sample treated with Na_2_CO_3_ showed no fluorescence intensity after background subtraction, while barely visible aggregation was observed. This can be explained by the fact that the alkaline conditions caused by Na_2_CO_3_ affected ThT, resulting in the loss of the ability to fluoresce (17).

We next tested the effect of Cu^2+^ on rPrP droplet formation, which is known to bind histidine residues in the N-terminal IDR region of PrP (18, 19). The samples containing CuSO_4_ did not show any droplet formation but had amorphous aggregates. These amorphous aggregates acquired slight ThT fluorescence after 24 h of incubation, suggesting that the aggregation was induced by the binding of Cu^2+^ to the N-terminal region (Fig. S3*A*). Furthermore, Cu^2+^ inhibited the SO_4_^2-^-induced droplet formation (Fig. S3*C*).

To examine the influence of pH, we tested at different pH values under conditions of 9%/9% of PEG/dextran with 120 mM Na_2_S_2_O_3_. At pH 4, a small number of spherical droplets were observed, but most of them formed ThT-positive, granule-like aggregates with a low circularity value. These granule-like aggregates did not fuse with each other. Among the conditions we tested, the droplets were most efficiently formed at neutral pH, although we could not fully evaluate the formation efficiencies at pH 12 because of the loss of ThT fluorescence (Fig. S3*D*). We confirmed that 120 mM Na_2_S_2_O_3_ and neutral pH were the optimal conditions for our experiments.

### The droplets of rPrP undergo liquid–solid phase transition

**Figure 2:**
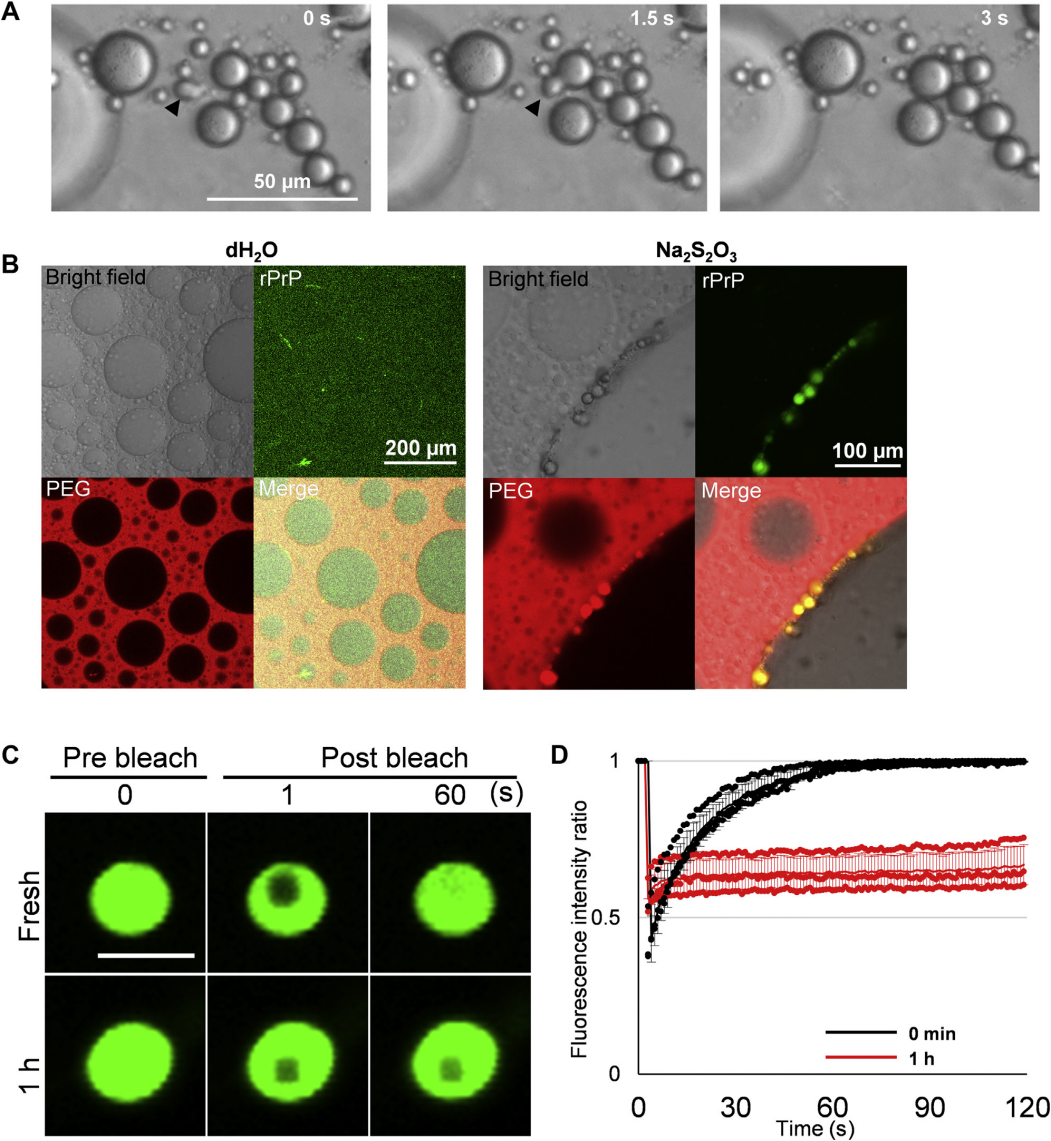
rPrP droplets undergo liquid–solid phase transition. *A*, consecutive images of droplets seamlessly combining with each other. The *black arrowhead* indicates a droplet uniting with another one. *B*, confocal microscopic images of distribution of PEG (0.01% of Rhodamine-PEG, *red*) and rPrP (1:18 of Alexa488-labeled rPrP, *green*). *Left panel*: Aqueous two-phase system/rPrP solution with dH_2_O. The scale bar represents 200 μm. *Right panel*: Aqueous two-phase system/rPrP solution with 120 mM of sodium thiosulfate. The scale bar represents 100 μm. Both images were acquired immediately after mixing the polymer and rPrP solution. *C*, confocal microscopic images from fluorescence recovery after photobleaching experiment of a fresh (0 min) droplet (*top*) and a droplet incubated for 1 h (*bottom*). The scale bar represents 5 μm. *D*, Fluorescence recovery after photobleaching curves from fresh droplets (0 min) and droplets incubated for 1 h regarding in regard to Fig. 2*C*. Each dot indicates a value measured in three independent experiments. Line indicates average value. Bar: SD. N = 3. rPrP, recombinant prion protein

To investigate the properties of the droplets, we continuously observed their behavior. The nascent droplets floating at the interface seamlessly fused with each other, suggesting that the droplets were in the liquid phase (Fig. 2*A*). Furthermore, rPrP immediately condensed to form droplets at the PEG/dextran interface by adding Na_2_S_2_O_3_ (Fig. 2*B*). In addition, fluorescence-labeled PEG colocalized with rPrP in the droplets, suggesting that PEG was bound to rPrP. These results clarified that the spherical structures without Alexa488 or ThT fluorescence in the background were polymer droplets in ATPS. Next, we conducted fluorescence recovery after photobleaching to the droplets, before and after 1 h of incubation. Fresh droplets, immediately after LLPS (0 min), showed full recovery of the intensity within 60 s after photobleaching, whereas the droplets incubated for 1 h at 37 °C showed no recovery throughout the observation period (Fig. 2, *C* and *D*), suggesting that the droplets of rPrP underwent liquid–solid phase transition and became rPrP-gels. However, it would be more important to investigate structural differences between droplets of rPrP formed in the initial stage and the aged gels. We failed to isolate the fresh droplets from ATPS because the fresh droplets became solid gels during centrifugation. Thus, the limitation of these experiments was that we could not directly compare the structure of liquid droplets formed in the initial stage and those in the solid gels post centrifugation.

### The N-terminal region of rPrP (residues 23–89) drives liquid–liquid phase separation and liquid–solid phase transition

**Figure 3:**
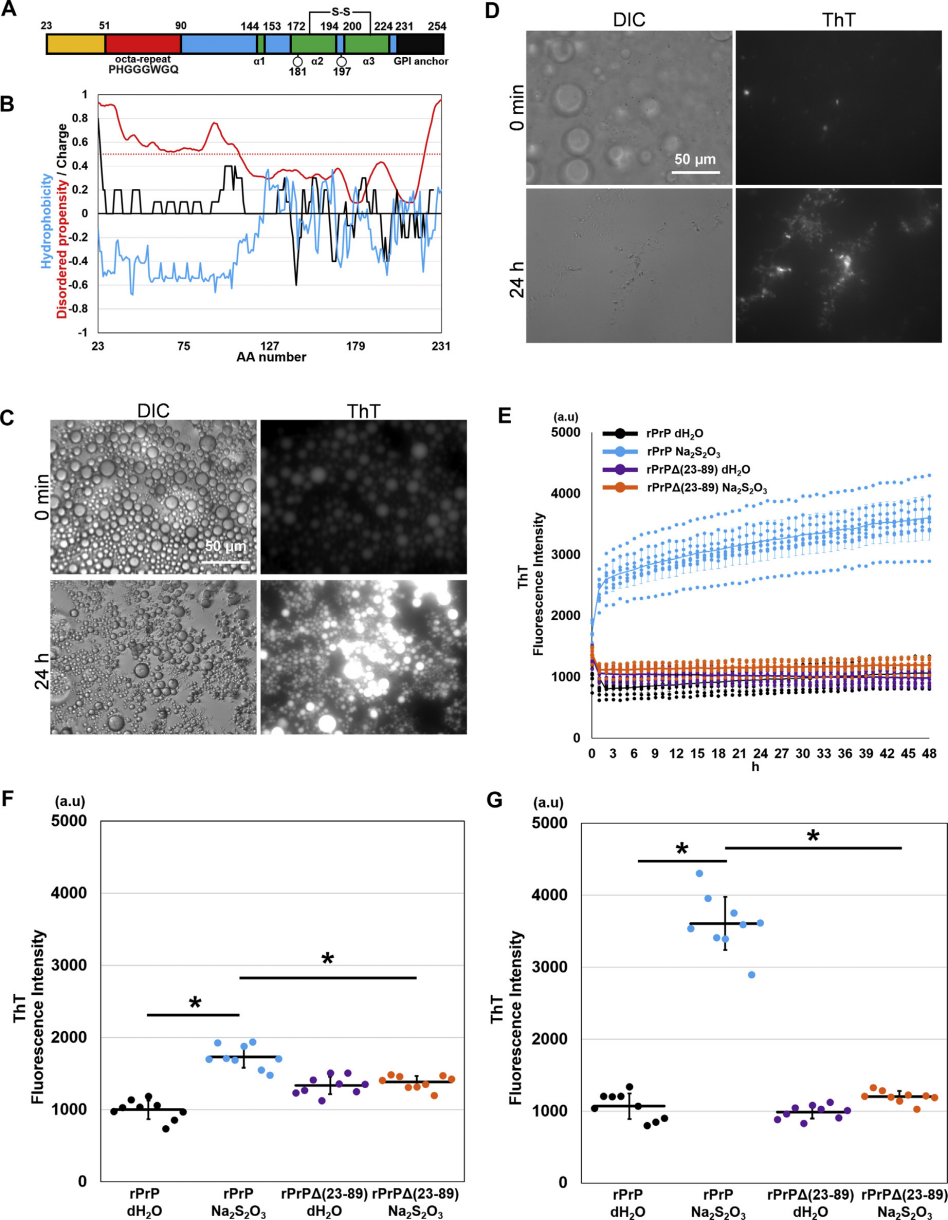
The N terminus of PrP is essential for droplet formation and maturation. *A*, schematic diagram of human rPrP residues 23 to 231. *Red* indicates octapeptide repeats region. *Green* indicates α helix regions. *Circles* indicate glycosylation sites. *B*, calculation of hydrophobicity, disordered propensity, and electric charge of human prion protein using Protscale, PrDOS, and EMBOSS. *Blue line*: hydrophobicity calculated by Protscale. *Black line*: electric charge calculated by EMBOSS. *Red line*: disordered propensity calculated by PrDOS. *Red dotted line*: threshold of disordered propensity (FP: 5%). *C*, DIC and fluorescence microscopic images of rPrP treated with Na_2_S_2_O_3_ at 0 min and 24 h. *D*, DIC and fluorescence microscopic images of rPrP Δ (23–89) treated with Na_2_S_2_O_3_ at 0 min and 24 h of incubation, acquired with long exposure. *E*, ThT fluorescence intensity of rPrP and rPrP Δ (23–89) measured in 48 h. Each *dot* represents a value measured in three independent experiments. *Line* indicates average of each group. *F* and *G*, quantification of ThT fluorescence intensity at 0 min and 48 h. ∗*p* < 0.0001. Bar represents SD. N = 9. Statistical analysis was performed using one-way ANOVA, followed by the Tukey–Kramer test. DIC, differential interference contrast, rPrP, recombinant prion protein; ThT, Thioflavin T.

The N-terminal region of PrP^C^ is known to be an IDR, whereas its C-terminus consists of stable secondary structures with three α-helices, as revealed by a nuclear magnetic resonance study, consistent with the prediction result from protein disorder prediction server (PrDOS) (20, 21). Under biological conditions, PrP^C^ is not phosphorylated or methylated but is a glycosylphosphatidylinositol-anchored protein with two glycosylation sites (Fig. 3*A*). To determine whether the IDR of rPrP influences LLPS, we first calculated its disordered propensity, hydrophobicity, and electric charge (Fig. 3*B*). This region coincides with the positively charged region predicted by EMBOSS and the hydrophilic region calculated from Protscale (22, 23, 24). In order to examine the molecular interactions, we calculated the possibility of planar π–π interactions (propensity score: PScore) by using an algorithm written by Venon *et al.* (25). The results showed that the residues from 23 to 104 were above the confidence threshold, which is defined as the possibly significant enrichment (>5- to 50-fold) of the π–π interaction (Fig. S4*A*). To elucidate the role of the N-terminal region, we compared the behavior of the full-length rPrP and N-terminally truncated mutant, rPrP Δ (23–89) in ATPS. We found that rPrP Δ (23–89) did not increase the fluorescence intensity even with Na_2_S_2_O_3_; however, it formed slightly ThT-positive aggregates at the interface. These aggregates showed no increase in ThT fluorescence throughout the observation period of up to 48 h, whereas the droplet of full-length rPrP increased the fluorescence intensity over time (Fig. 3, *C*–*E*). Even very small droplets (<5 μm) with no apparent ThT fluorescence at 0 min could be clearly identified after 24 h of incubation. Furthermore, the fluorescence intensity was significantly higher than that of rPrP Δ (23–89) with Na_2_S_2_O_3_ at 1 h and it became more striking after 48 h (Fig. 3, *F* and *G*). Full-length mouse rPrP (Mo–rPrP residues: 23–231) also showed similar results (Fig. S4, *B* and *C*).

### Liquid–solid phase transition involves conformational conversion of the prion protein

**Figure 4:**
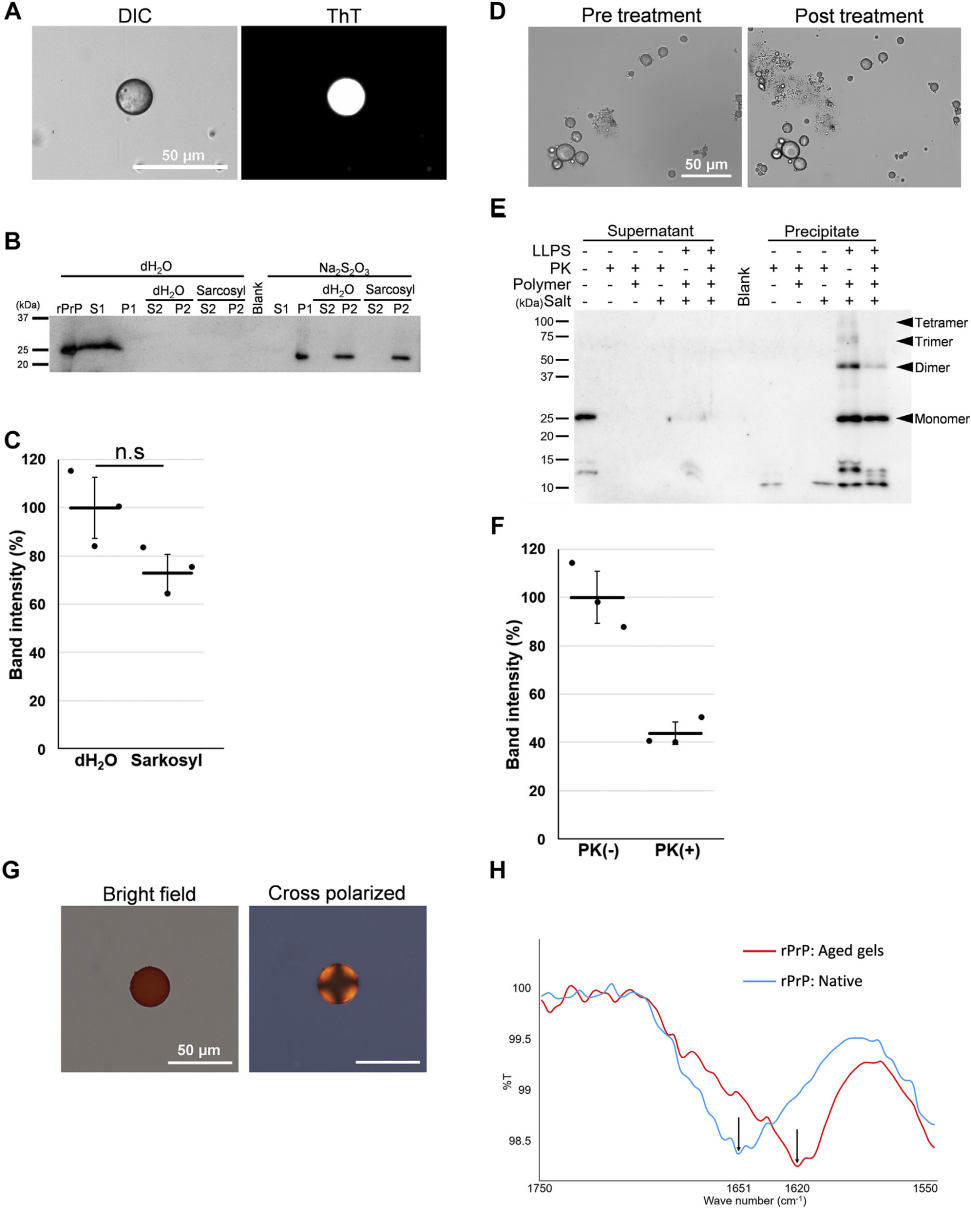
Biochemical analysis of the rPrP-gel. *A*, DIC and fluorescence microscopic images of the rPrP-gel incubated for 30 min in aqueous two-phase system, collected by centrifuge, and then applied into dH_2_O with 50 μM of ThT. The scale bar represents 50 μm. *B*, western blotting of rPrP, with or without sarkosyl treatment. S1 and P1 were originally collected by centrifugation from the sample diluted with dH_2_O. S2 and P2 were collected from the P1 fraction treated with dH_2_O or sarkosyl. *C*, quantification of band intensity from P2 fraction of dH_2_O compared with sarkosyl treatment (refer to Fig. 4, *B* and *D*). *D*, DIC microscopic images of aged gels before (0 min) and after (30 min) PK treatment. *E*, western blotting of rPrP aged gels. “LLPS” indicates that experiments were done under the condition of 9%/9% of PEG/dextran, 120 mM of sodium thiosulfate, and 10 μM of rPrP. “dH_2_O” indicates that experiments were done under the condition of 9%/9% of PEG/dextran, 10 μM of rPrP without salt. “Polymer” and “Salt” indicates that the samples contain 9%/9% of PEG/dextran and 120 mM of sodium thiosulfate. “PK” indicates that samples were treated with 7.5 μg/ml of PK. Each *dot* represents a value measured from individual sample. *Solid line* indicates average. Bar, SD. N = 3. Statistical analysis was performed with unpaired *t* tests. “n.s.” means no significant difference. *F*, quantification of band intensity of aged gels, with or without PK treatment (refer to Fig. 4*E*). *G*, confocal microscopic images of aged rPrP-gels stained with Congo-red in bright field and cross-polarized. The scale bar represents 50 μm. *H*, FTIR spectroscopic analysis of aged rPrP-gels and native rPrP. *Red line*: the aged rPrP-gel. *Blue line*: native rPrP. *Arrows* indicate the peak of each sample. DIC, differential interference contrast, PK, Proteinase K; rPrP, recombinant prion protein; ThT, Thioflavin T. *Solid line* indicates average. Bar, SD. N = 3. Statistical analysis was performed with unpaired *t* tests. “n.s.” means no significant difference.

Because the rapid growth of ThT fluorescence coincides with the timing of a liquid–solid phase transition (Fig. 2, *C* and *D*), we investigated how aging affects the biochemical properties of the rPrP-gels. First, we aged the droplets for 30 min and then collected them by centrifugation. First, we aged the droplets for 30 min and then collected them by centrifugation. The rPrP-gels were ThT positive and did not dissolve in water (Fig. 4*A*). Subsequently, we resuspended the gels in 1% sarkosyl and reprecipitated them by centrifugation. Western blot analysis showed that rPrP was insoluble in 1% sarkosyl solution (Fig. 4*B*). There was no significant difference in the insoluble fraction (P2), with or without treatment (Fig. 4*C*). Next, we examined whether these PrPs in the gels acquired PK resistance. We aged the rPrP-gels for 72 h and then digested them with PK. The appearance of aged gels remained unchanged after PK digestion (Fig. 4*D*). SDS-PAGE and Western blotting of the aged gels collected by centrifugation showed that the aged droplets contained oligomers of rPrP and 40% of rPrP remained undigested (Fig. 4, *E* and *F*). A small PrP-res fragment was detected around 10 to 15 kDa. We could not disrupt the aged gel by sonication to improve the penetration of proteinase. There was no significant difference in PK resistance between the aged rPrP-gel and brain-derived PrP^Sc^, even though they showed different band patterns (Fig. S4, *D* and *E*). We further attempted to confirm that the rPrP-gel was composed of amyloid. The aged gels stained positively with Congo red, although they did not show apple-green birefringence under cross-polarized light (Fig. 4*G*). A similar observation was reported in human amyloid spherulites composed of islet amyloid polypeptide in the pancreatic tissue of type 2 diabetes mellitus (26). However, the aged gel did not show the Maltese cross under cross-polarized light, which is a characteristic of spherulites. To analyze the secondary structure of the aged gel, we performed FTIR analysis. FTIR analysis showed that the aged gels had a distinctive peak at 1620 cm^−1^ in the β-sheet region of the second-derivative spectra, which shifted from 1651 cm^−1^ in the α-helix region from the native form of rPrP (Fig. 4*H*). Moreover, they were stable for months in water (data not shown). These results suggest that rPrP is converted into PrP-res inside the droplets, acquiring a β-sheet–rich structure, detergent insolubility, and PK resistance. We next investigated whether the aged rPrP-gel has seeding activity. The aged rPrP-gels were subjected to real-time quaking-induced conversion (RT-QuIC) analysis (Fig. S4*F*). As a result, the samples with the rPrP-gels added as seed did not show ThT positivity (Fig. S4*F*), and the rPrP-gels maintained its shape even after 100 cycles of the reaction, indicating that rPrP-res generated from the rPrP-gels did not have seeding activity.

## Discussion

We have demonstrated that rPrP undergoes LLPS at the interface of the ATPS. IDR in the N-terminal region of PrP^C^ (residues 23–89) and kosmotropic anions were essential for the overall reaction. The rPrP liquid droplets subsequently showed liquid–solid phase transition within an hour, and the aged rPrP-gels contained β-sheet–rich, sarkosyl-insoluble, and PK-resistant amyloid.

ATPS has been used for a wide range of purposes, such as purification of enzymes, nucleic acids, and viruses to stabilize their structure (14, 27). The partitioning behavior in ATPS has been well documented. In protein purification, monomeric IgG could be collected separately in the PEG-rich fraction because of its positive charge (28). Such convention by ATPS can facilitate interactions of the sequestered molecules, as demonstrated by DNA and actin fibers separately interacting and polymerizing inside the dextran phase, called cell-sized aqueous microdroplets, imitating cell-like crowded microenvironments (29). Similarly, our present results could be interpreted from a similar viewpoint. It is conceivable that rPrP, which has positive charges like IgG in the IDR, was sequestered to the PEG phase at first and then, when it was sufficiently condensed, formed a liquid phase on its own owing to the interactions between the IDRs. Similar to many other proteins known to undergo LLPS, the region consists of five repeats of a glycine-rich motif and contains proline and aromatic amino acids such as tryptophan. However, it is not clear why the interface of ATPS is the essential factor for inducing LLPS of rPrP. The hydrophobicity of the protein surface and solubility in different polymer solutions may affect the behavior of the protein in ATPS (14, 30). Since little is known about protein behavior in ATPS, further investigation is required to answer this question. It is unlikely that the fluorescence-labeled PEG colocalized with the droplet of rPrP directly induces the reaction of rPrP because it hardly affects the conversion properties of PrP^C^ to PrP-res (31).

Proline and glycine-rich N-terminal IDRs of rPrP are very flexible and multivalent because of the periodically located tryptophan residues; these features enable efficient intermolecular interactions and consequently LLPS. The liquid phase formation *via* IDR may subsequently facilitate interactions between the C-terminal regions and finally evoke parallel β-sheet conversion of the entire molecule. Therefore, it does not require agitation to catalyze the reactions. This may be in contrast to the facilitation of conversion by mechanical agitation, where natively folded protein molecules at the air–water interface are denatured and the forcefully exposed hydrophobic residues presumably enable efficient intermolecular binding, and eventually conversion (32, 33, 34).

The kosmotropic anions have been shown to stabilize the structure of proteins and enhance amyloid formation *in vitro*. Originally, it was described as the Hofmeister series, which is a series of anions and cations to order the relative protein salting-out abilities (35, 36). It has been shown that the efficiency of amyloid formation from prion protein is in accordance with the Hofmeister series (37, 38). Kosmotropic anions have been shown to drastically improve the detection limit of pathological amyloids, including prions (39). However, the reason why the efficiency of droplet formation does not exactly match with the Hofmeister series is unclear. Copper ions are well known to bind to the N-terminal region of prion proteins and play an important role in the aggregation process (40, 41). Cu^2+^ has been shown to affect the structural conversion of both rPrP and PrP^C^ into proteinase-resistant aggregates (42, 43). It has been shown that this aggregation process is triggered by binding between PrP and Cu^2+^ (44). Because Cu^2+^ binds to the N-terminal region in histidine residues, Cu^2+^ affects its flexibility and initiates conformational changes (45, 46). Therefore, our result that rPrP was unable to undergo LLPS in the presence of Cu^2+^ can be explained by the loss of flexibility of the N-terminal region due to Cu^2+^ and rPrP binding. Considering these previous findings and our data, kosmotropic anions may interact with the N-terminal region of prion protein as well as Cu^2+^. These ions may work differently in the conversion process: (i) metal binding cations induce direct conversion from soluble rPrP into solid aggregates, or (ii) kosmotropic anions induce liquid–liquid phase separation and sequential phase transition.

**Figure 5:**
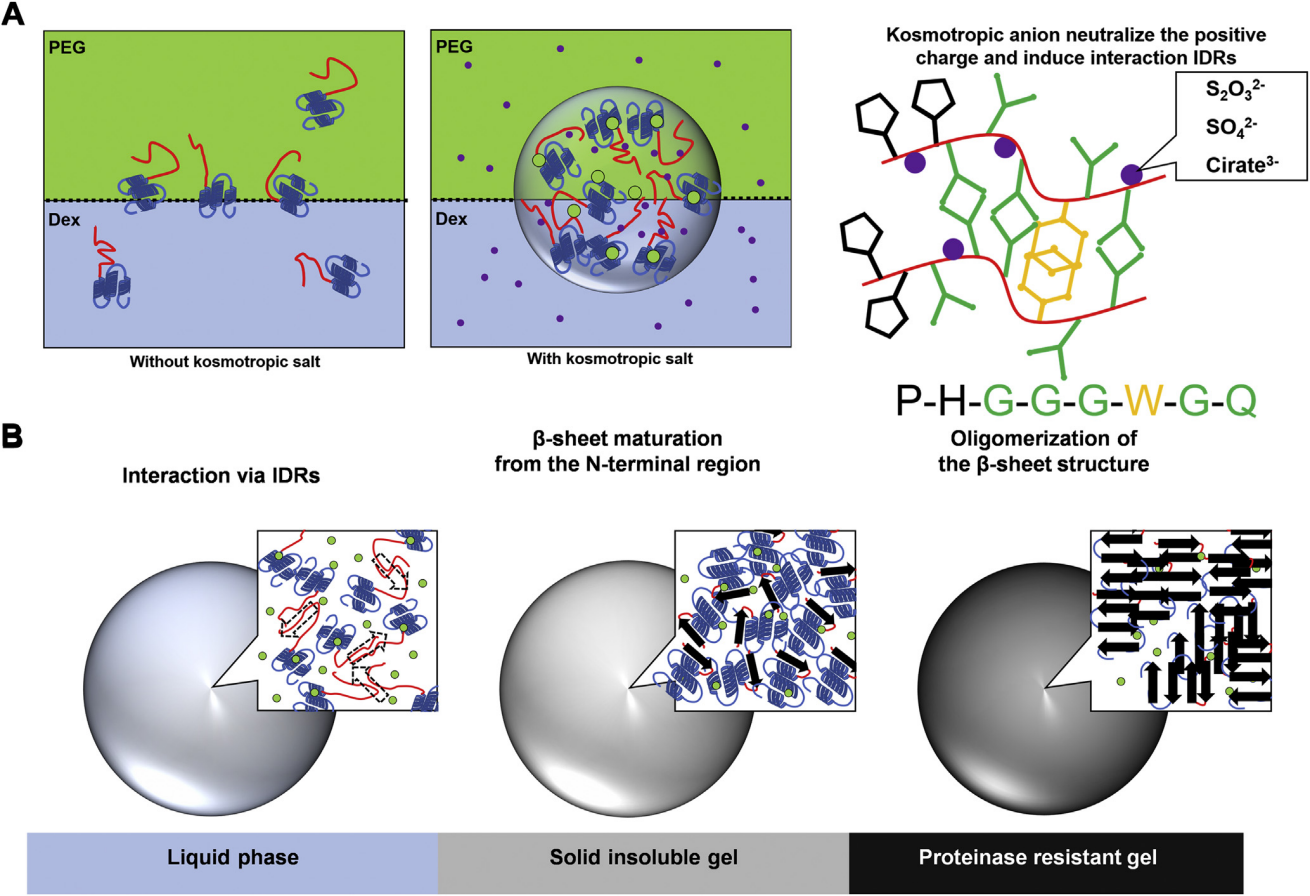
Hypothetical model for droplet formation and phase transition. *A*, *left*: Prion protein molecules are equally dissolved in PEG (*yellow green*) and dextran (*blue*) fractions without kosmotropic anions. *Middle*: Prion protein molecules assemble each other *via* IDRs and form droplets at the interface of the aqueous two-phase system by adding kosmotropic anions (*purple dots*). PEG may bind to prion protein but with no change in its structure. *Right*: possible IDRs (*red line*) interaction inside droplets; kosmotropic anions neutralize the positive charge of IDRs and induce interaction between them. Dipole–dipole interaction (*green*) of glycine (G) and glutamine (Q), and π–π interaction (*yellow*) of tryptophan (W) are expected to underlie in phase separation and transition (refer to (52)). *B*, hypothetical model of liquid–solid phase transition. In a fresh droplet, the IDRs of prion protein may construct a cross β-sheet structure (*dotted arrow*). As the droplet matures, β-sheet conversion is initiated from IDRs, forming an insoluble gel. Finally, the entire molecule is converted to β-sheet–rich structure and oligomerized, resulting in proteinase-resistant gel. *Yellow green*, PEG fraction and PEG molecule. *Light blue*, dextran fraction. *Red*, IDRs of rPrP. *Dark blue*, constructed region of rPrP including three α helices. *Purple dots*, kosmotropic anions. *Arrows*, β-sheet structure. IDR, intrinsically disordered region.

IDR has been shown to assemble protein molecules and form a cross-β structure comprising stacks of short β-strands in the process of liquid-solid phase transition (47). Further investigation is required to elucidate the process of β-sheet conversion. It is well described about the importance of protein–protein interactions, especially the proteins that are reported to undergo LLPS. Of note, π–π or π–cation interactions between aromatic residues are considered to play a major role in LLPS and phase transition (48, 49, 50, 51). IDRs of PrP, that is, octapeptide (PHGGGWGQ) repeats, are very flexible and multivalent; thus, the octapeptide region may quickly form a short cross-β structure, as suggested by the weak ThT fluorescence seen at the very beginning of droplet formation. Inside the droplet, the flexible intermolecular interactions of rPrP through the octapeptide repeats maintain the C-terminal regions of rPrP in proximity of each other until they may be fully converted into β-sheet–rich structures. In addition, repeats of the motif are expected to have advantages in the liquid phase because of the high plasticity of intermolecular bindings under shear stress. In summary, we propose the following hypotheses: (i) the N-terminal region with positive charges induces condensation of rPrP in the PEG phase; (ii) the charges are neutralized by the kosmotropic anion, inducing direct interaction of the dipole (G,Q) and aromatic residue (W) of the octapeptide region to form a short cross-β-sheet structure (39); and (iii) the molecular distance of the C-terminal region is reduced, enabling them to become close to each other, leading to the polymerization and β-sheet conversion of the entire rPrP to the amyloid. (Fig. 5, *A* and *B*). This process may be similar to the *in silico* simulation model, suggesting that the conversion process started from the N-terminal region (53). It has been shown that TDP-43 LLPS is mediated by a few aromatic residues (54). Further experiments are required to identify the role of specific amino acids in the N-terminal region in LLPS.

Although our experimental conditions were highly artificial, it is still worthy to consider the possibility that the LLPS of PrP^C^ could occur *in vivo*. LLPS is a phenomenon that was initially reported for intracellular proteins; however, later, proteins associated with the cell membranes were also shown to undergo LLPS. Recently, it has been reported that zona occludens, a membrane-associated scaffolding protein, underwent LLPS to form functional tight junctions between cells (55), suggesting that the protein complex attached to the membrane certainly has the properties of liquid. PrP^C^ is anchored to the cell membrane and can interact with various macromolecules, including proteins, RNA, and lipids (56, 57). These macro-biomolecules are intertwined and might drive liquid–liquid phase separation of the membrane protein. Of interest, it has been demonstrated that Aβ-oligomers and DNA-aptamers drive the liquid–solid phase transition of rPrP through the interaction of amino acid residues around 90 to 120 (58, 59). Furthermore, both full-length prion protein and prion protein peptide (amino acid, 23–144) could form proteinase-resistant, spherical–ellipsoid aggregates that grow as amyloid fibrils by the addition of detergent or polysaccharides, thus supporting our hypothesis that liquid–solid phase transition is associated with prion diseases (60, 61, 62). However, given that several reports have successfully demonstrated the acquisition of seeding activity from rPrP/PrP^C^ by adding RNA, lipid, and other proteins (63, 64, 65), our results argue that the acquisition of PK resistance of rPrP from LLPS alone is not sufficient for seeding activity. We would like to note that it is not clear from our results whether rPrP in the state of droplets, nascent or very long-term aged gels is infectious. Further infection experiments in nonhuman primates are necessary to ensure safety, and the droplets/gels should be handled in accordance with potential prion.

Under physiological conditions, PrP^C^ is anchored at the cell surface *via* the glycosyl phosphatidyl inositol anchor. The degradation half-life for PrP^C^ has been shown to be several hours (66, 67). During metabolic process, 10% to 50% of PrP^C^ is cleaved at the amino acid between 111 and 112, which is called “C1 fragments,” and the N-terminal region is released into extracellular environments (68, 69), and the resident C1 fragments of PrP^C^ remains on the cell surface. A previous study showed that the C1 fragment has not been a substrate for conversion to PrP^Sc^ (70). However, it was reported that the full-length PrP^C^ internalized by fluid endocytosis returned to the cell membrane. In addition, full-length PrP^C^ has been detected in exosomes (71, 72, 73). A variety of biological macromolecules may form crowding conditions with multiple interfaces such as lipid rafts, exosomes, and endocytic vesicles. Of interest, PrP^C^–PrP^Sc^ conversion has been reported to occur in lipid rafts on the cell surface and endosomes (74, 75, 76, 77). The clustering formation of full-length PrP^C^ on the cell surface has been observed during PrP^C^–PrP^Sc^ conversion (78). These previous findings indicate the existence of LLPS in the conversion process. In such situations, interactions between multivalent and flexible IDR of PrP might further condense the molecules, restraining their motions and directions. Taken together, microenvironments *in vivo* with high concentrations of kosmotropic anions may drive LLPS of PrP^C^, leading to spontaneous intra- and/or extracellular PrP-amyloid formation. Further experiments using cell culture and *in vivo* imaging are needed to elucidate whether PrP^C^ can undergo LLPS *in vivo*.

LLPS of full-length rPrP using ATPS was demonstrated. The droplets of rPrP appeared only at the interface between PEG and dextran. The N-terminal region of prion protein (amino acids 23–89) and kosmotropic anions in neutral pH were essential for this reaction. The liquid–solid phase transition was found to be accompanied by β-sheet transition, resulting in PK resistance, although the aged rPrP-gel did not show seeding activity. We are not able to state at this point that LLPS is the mechanism by which prions are generated. Given the fact that β-sheet conversion along with liquid–solid phase transition converted rPrP into PrP-res (an amyloid albeit noninfectious), promoted by LLPS at the interface of macromolecules, we propose LLPS as a potential mechanism for the formation of infectious or pathological amyloids such as prions as well.

## Experimental procedures

### Protein expression and purification

We prepared three rPrPs: full-length human PrP (residues 23–231), truncated human PrP (residues 90–231), and Mo-rPrP (residues 23–231). All constructs were expressed in the *Escherichia coli* strain DH5α. The expression and purification of rPrPs were performed as described (9, 79). After purification, each protein solution was frozen at −80 °C in 150-μl aliquots, which were thawed for single use. Before an aliquot was used for any experiment, each protein solution was centrifuged at 21,880*g* for 10 min at room temperature (28 °C). To prepare labeled rPrP, an Alexa Fluor 488 Microscale Protein Labeling Kit (A30006, Invitrogen) was used. The procedure was performed in accordance with the manufacturer’s instructions.

### Disorder propensity and charge prediction

Disorder propensity was calculated using PrDOS (20), charge prediction was performed using EMBOSS (22), and the hydrophilic region was calculated using ProtScale (23, 24). π–π interaction was calculated using the phase separation propensity score (PScore) (25). The amino acid sequence from Uniprot (P04156) was used.

### Droplet formation assay (polymer and salt solution preparation)

Polymer solutions were prepared from polyethylene glycol (PEG) (MW, 6000) (Wako) and dextran (MW, 180,000) (Nacalai Chemical). Each component was dissolved in dH_2_O and prepared as 50% PEG and 25% dextran (w/v) and stored at 4 °C in 1 ml aliquots. The phase diagram was created by direct observation of polymer droplets using differential interference contrast (DIC) microscopy. PEG-dextran polymer solutions were prepared as 1% to 10% (w/v) of each polymer in 20 μl of solution. The polymer solutions were vigorously vortexed, and 5 μl of the solution was loaded onto a glass slide. For confocal microscopy observation with fluorescence, 0.01% rhodamine-PEG (#PG1-RB-5k, Nanocs) was used. For salt solutions, we prepared 2 M stocks of NaCl, Na_2_S_2_O_3_, Na_2_CO_3,_ CuSO_4_ (Wako), Na_3_C_6_H_5_O_7_, Na_2_SO_4_, and (NH_4_)_2_SO_4_ (Nacalai Chemical). Each solution was stored at room temperature. To prepare the ATPS solution, each polymer solution was mixed at concentrations ranging from 1.5% to 13.5% with 200 mM salt (final: 1–9 w/v % of each polymer, PEG/dextran with 120 mM salt).

Then, the solution was mixed well by pipetting and vigorously vortexed. Experiments were performed on a scale of 50 μl (52.6 μl with ThT); 2.6 μl of 1 mM ThT (final concentration, 50 μM) was added to 30 μl of ATPS solution, and then 20 μl of rPrP solution (final concentration, 2–10 μM) was added to the ATPS solution and gently pipetted 10 to 15 times. The entire solution was applied to a glass slide or 96-well plate (#165305, Thermo Fisher Scientific) for microscopic observation. Droplet observations were performed using confocal microscopy (#LMS700; Carl Zeiss) and DIC microscopy (Axioskop2; Carl Zeiss) with 20x and 40x objective lenses. To evaluate ThT fluorescence, Colibri seven (Carl Zeiss) was used as a luminous source at a wavelength of 485 nm. Images were acquired with exposures of 250 (low exposure), 500, and 2000 ms (high exposure). We detected rPrP droplets by ThT or Alexa 488 fluorescence. The pH was adjusted using NaOH (1 mol/l) or HCl (1 mol/l) and confirmed by test paper. For droplet aging, the samples were applied to a 96-well plate or Eppendorf tube incubated at 37 °C for 30 min to 72 h. All experiments were performed in triplicate.

### Congo red staining

The samples were incubated for 72 h at 37 °C in the ATPS solution. After incubation, 200 μl of dH_2_O was added and mixed well by pipetting. The aged rPrP-gels were collected by centrifugation at 21,800*g* for 10 min at room temperature (26–28 °C) and were stained with 50 μl of 1% Congo red (#C8,445-3, Aldrich) solution for 30 min in an Eppendorf tube at room temperature. After staining, the sample was centrifuged again under the same conditions, and the supernatant was discarded. The pellet was washed with 50 μl of dH_2_O by pipetting, the solution was centrifuged again under similar conditions, and the supernatant was discarded. The pellet was suspended in 20 μl of dH_2_O, and 5 μl was applied on a glass slide and sealed with a cover glass. Microscopic observation was performed using a confocal microscope (Nikon) with a polarization filter. Images were acquired using the NIS-Elements C software.

### Fluorescence after photobleaching assay

Fluorescence recovery assay after photobleaching was performed using LMS700. Alexa488-labeled human rPrP was diluted 1:18 with native human rPrP (final concentration, 13 μM). Bleaching was performed with 100% transmission of a 405-, 488-, or 500-nm laser. Prebleaching images were taken for 3 s (1-s frame rate, 3 frames), whereas postbleaching images were acquired for the following 120 s (1-s frame rate, 120 frames) and analyzed with ZEN. The samples named “0 min” were taken in less than 5 min, including the setup. The sizes of the bleached area and background area were set in the first experiment. For each image, the bleached region and background region were calculated using ZEN, and the background was subtracted during analysis.

### Sarkosyl and proteinase K treatments

Sarkosyl and PK were purchased from Sigma-Aldrich. For sarkosyl treatment, the sample was incubated in the ATPS solution at 37 °C for 30 min, and 200 μl of dH_2_O was added to the sample and mixed well by pipetting. The entire solution was centrifuged at 21,880*g* for 10 min at room temperature. Supernatant-1 (S1) and Pellet-1 (P1) were collected. P1 was suspended in 25 μl of dH_2_O or 1% sarkosyl and incubated at 37 °C for 10 min. After incubation, samples were centrifuged at 21,880*g* for 30 min at room temperature (26–28 °C) and then Supernatant-2 (S2) and Pellet-2 (P2) were retrieved. The PK solution (10 μg/ml) was prepared in dH_2_O. The samples were incubated at 37 °C for 72 h in an Eppendorf tube. As a negative control, the solution containing the same amount of rPrP was treated with the PK solution. The samples and PK solution were mixed by pipetting, applied to a 96-well plate, and incubated at 37 °C. DIC microscopy was performed at the beginning of the reaction (0 min) and at the end of incubation (30 min). Samples were retrieved from the 96-well plate, and each well was washed with 100 μl of dH_2_O. The entire sample was collected in an Eppendorf tube and centrifuged at 21,880*g* for 10 min at room temperature (26–28 °C). The supernatant and pellet were collected. In both experiments, the supernatant was denatured with 6x SDS sample buffer (50 mM Tris-HCl pH 6.8, 5% glycerol, 1.6% SDS, and 100 mM dithiothreitol). The pellet was then resuspended in 1x SDS buffer and boiled at 95 °C for 10 min for SDS-PAGE.

### Brain homogenate preparation

BHs from 22L and sporadic Creutzfeldt-Jakob disease-infected mice were prepared as described (80, 81). BH (20% [w/v]) was mixed with 2x lysis buffer (100 mM Tris-HCl [pH 7.5] containing 300 mM NaCl, 1% Triton X-100, 1% sodium deoxycholate, 4 mM EDTA, and protease inhibitors) and incubated at 4 °C for 30 min and then centrifuged at 9000*g* for 5 min. The supernatant was collected and protein concentration was measured using a BCA protein assay kit (#23227 Thermo Fisher Scientific). PK treatment was performed at a ratio of protein to enzyme from 1:10, 1:50, 1:100, and 1:200, respectively. A total of 65 μg of protein from BH was loaded onto SDS-PAGE.

### Immunoblotting

Samples were loaded onto an 18% acrylamide gel for SDS-PAGE and then transferred to a polyvinylidene difluoride membrane. The membrane was blocked using 5%(w/v) skim milk with TBST (10 mM Tris-HCl pH 7.8, 100 mM NaCl, 0.1% Tween 20) at RT for 1 h. To detect PrP, the membrane was incubated with primary antibody R20 (1:1000 diluted with 1% skim milk) for 1 h at room temperature (26–28 °C) (82). Horseradish peroxidase–conjugated anti-rabbit IgG (1:10,000, GE Healthcare Life Sciences) was used as the secondary antibody. Protein bands were visualized using Clarity Western ECL substrate (Bio-Rad). The band intensity was quantified using ImageJ software.

### Quantifying ThT fluorescence

The fluorescence intensity was quantified with FLUOstar Omega (BMG Labtech) in a 96-well plate with a spiral scan. The 96-well plate was covered with sealing tape (#J676060, Greiner), incubated at 37 °C, and monitored by the bottom reading of the fluorescence intensity every hour up to 48 h using monochromators or filters with wavelengths of 448 (excitation) and 482 nm (emission).

### Fourier transform infrared spectroscopy analysis

Fourier transform infrared spectra were measured using a JASCO FT/IR-4700ST with attenuated total reflection. Five microliters of the sample was loaded onto the grid. To prepare the sample for FTIR, we first prepared a 30x concentrated sample (aged for 72 h) from 1.5 ml scale to 50 μl. The aged gel was collected by centrifugation, as described above, and suspended in dH_2_O. Recombinant PrP (residue 23–231, 20 mg/ml) with normal refolding after purification was used as control “native PrP.”

### Evaluation of seeding activity

Seeding activity was investigated using RT-QuIC as described (9). The aged rPrP-gel of hu-PrP was retrieved after 72 h of incubation. Five of 50 μl ATPS solutions treated with Na_2_S_2_O_3_ was centrifuged as described above, and the collected precipitates were gathered into a single tube with 50 μl of dH_2_O. The rPrP-aged gel solution (1x) was serially diluted to 10^-1^ and 10^-2^ solution with dH_2_O, and a 10-μl aliquot was added to each well as a seed. Homogenates from sporadic Creutzfeldt-Jakob disease-infected or normal brain from knock-in mice were serially diluted to a final concentration of 1 × 10^-3^ (w/v), and then 10-μl aliquots were used as positive and negative controls.

## Data availability

All data are contained within the article.

## Supporting information

This article contains supporting information (25).

## Conflict of interest

The authors declare that they have no conflicts of interest with the contents of this article.
