# Supplementary material for: Liquid–liquid phase separation of full-length prion protein initiates conformational conversion in vitro
Source: J Biol Chem. 2021 Feb 2;296:100367. doi: 10.1016/j.jbc.2021.100367 (PMC8289115; doi:10.1016/j.jbc.2021.100367)
Supplement: Figures S1–S4 [file mmc1.pdf]

## S-1

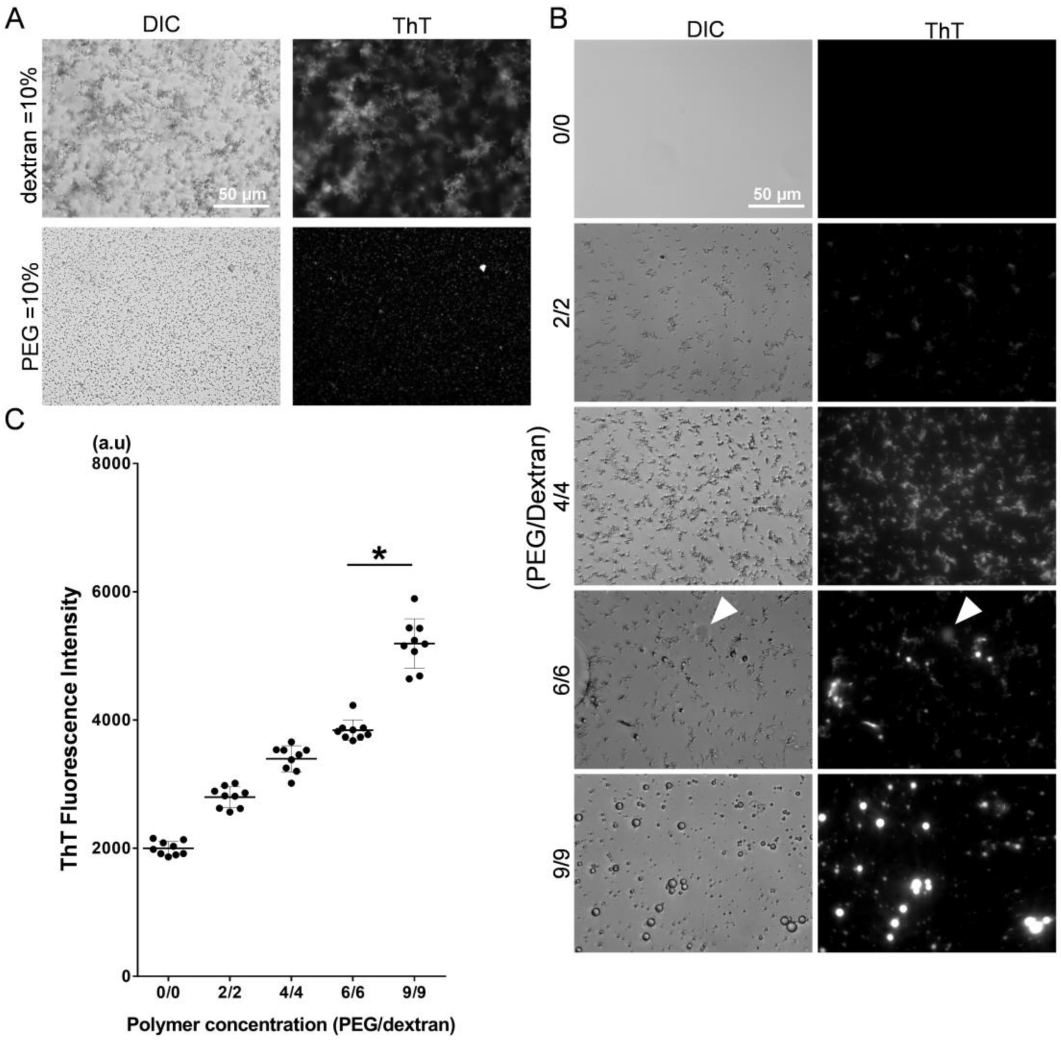


**Sup. Fig. 1 rPrP undergoes liquid phase separation above the binodal curve of ATPS.**

(A) DIC and fluorescence microscopic images of rPrP (10 µM) mixed with 10% dextran (Top) or 10% PEG (bottom) with 120 mM thiosulfate sodium. rPrP salted out as a ThT-positive aggregate.

(B) DIC and fluorescence microscopic images of the bottom of the well after 24 h of incubation are shown in Fig. 1B. ThT-positive amorphous aggregates precipitated in 2% –4%/2% –4%, whereas slightly ThT-positive puddle-like droplets (white arrowhead) and amorphous aggregation coexisted with spherical-shaped precipitates in 6%/ 6%, and only spherical precipitates were observed in the 9%/ 9% condition.

(C) Univariate scatterplot for ThT fluorescence intensity of each polymer concentration after 24 h of incubation. Each dot indicates a value measured in 3 independent experiments. The thin line shows SD, and the bold line shows the average. N=9. *$P < 0.0001$. Statistical analysis was performed using one-way ANOVA, followed by the Tukey-Kramer test.

**S-2**

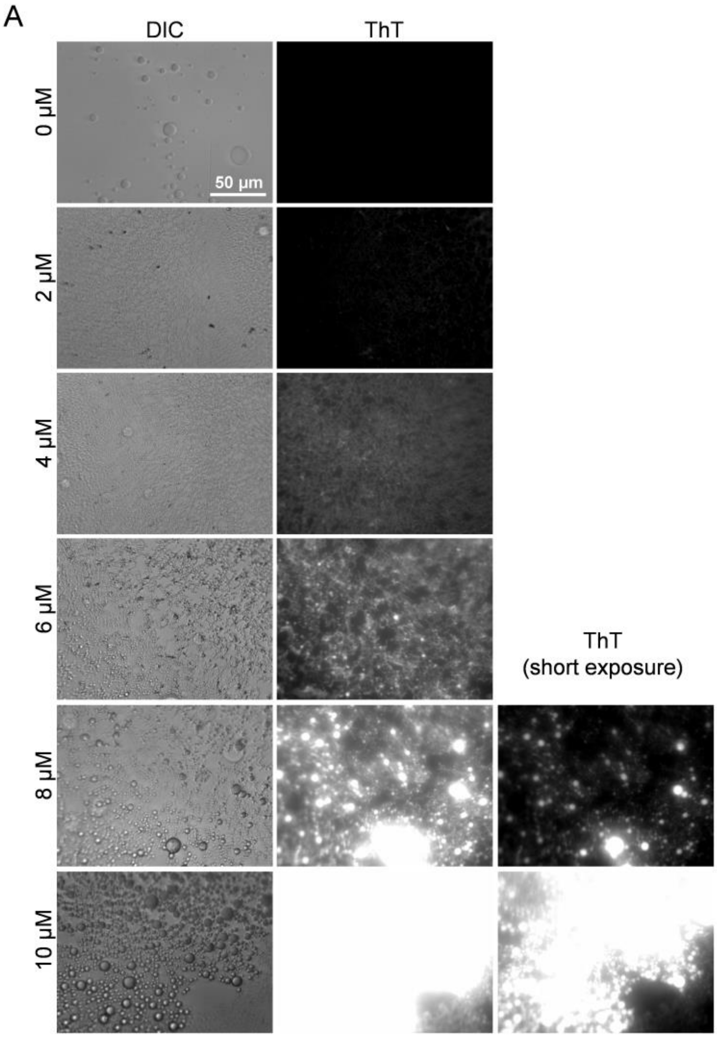

**S-2**

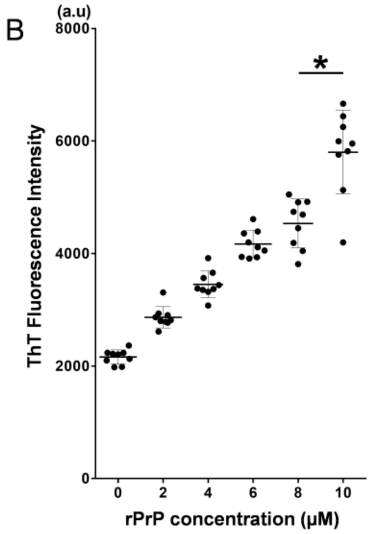


**Sup. Fig. 2 Droplet formation efficiency corresponding to rPrP concentration.**

(A) DIC and fluorescence microscopic images of rPrP at each concentration after 24 h of incubation.
(B) Univariate scatterplot of ThT fluorescence intensity for each rPrP concentration after 24 h of incubation. Each dot indicates a value measured in 3 independent experiments. The thin line shows SD, and the bold line shows the average. N=9. *$P < 0.0001$. Statistical analysis was performed using one-way ANOVA, followed by the Tukey-Kramer test.

**S-3**

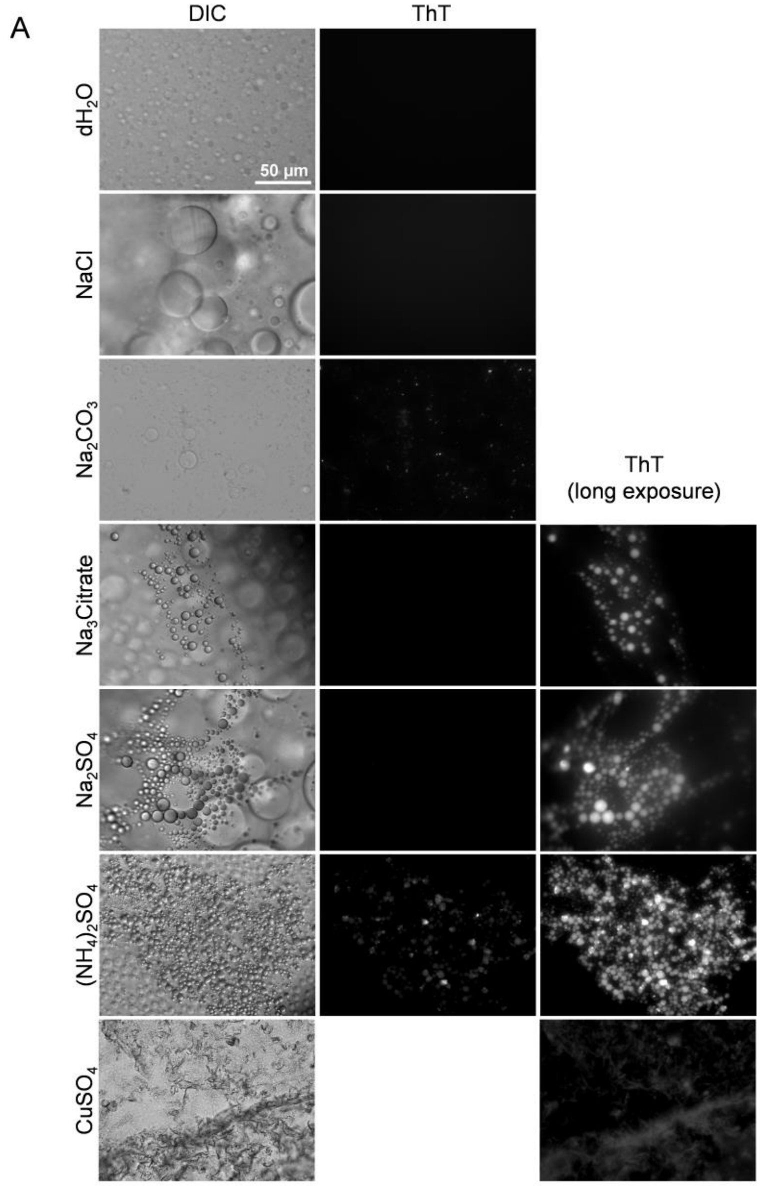


**Sup. Fig. 3 Effect of salts and different pH values on droplet formation.**

(A) DIC and fluorescence microscopic images of the interface of wells after 24 h of incubation with various salts. Left: DIC. Middle: fluorescence microscopic images Right: Acquired with high exposure.

**S-3**

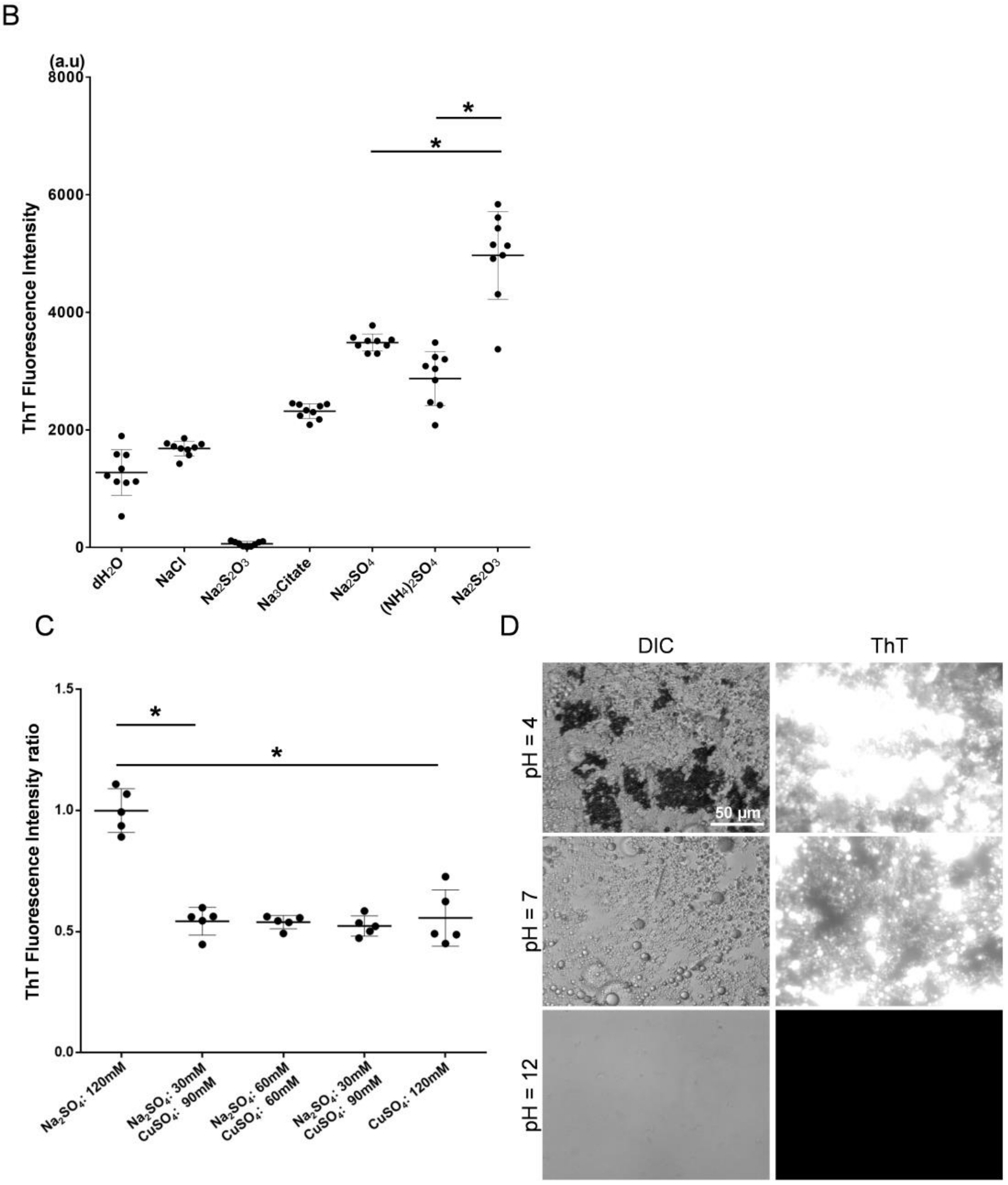


(B) A univariate scatterplot of the fluorescence intensity according to the salts. Each dot indicates a value measured in 3 independent experiments. The thin line shows SD, and the bold line shows the average. N=9. *$P < 0.0001$. Statistical analysis was performed using one-way ANOVA, followed by the Tukey–Kramer test.

(C) A univariate scatterplot of the fluorescence intensity according to the concentration of $Na_2SO_4$ and $CuSO_4$. The thin line shows SD, and the bold line shows the average. N=5. *$P < 0.001$. Statistical analysis was performed using one-way ANOVA, followed by the Tukey–Kramer test.

(D) DIC and fluorescence microscopic images at each pH after 24 h of incubation.

**S-4**

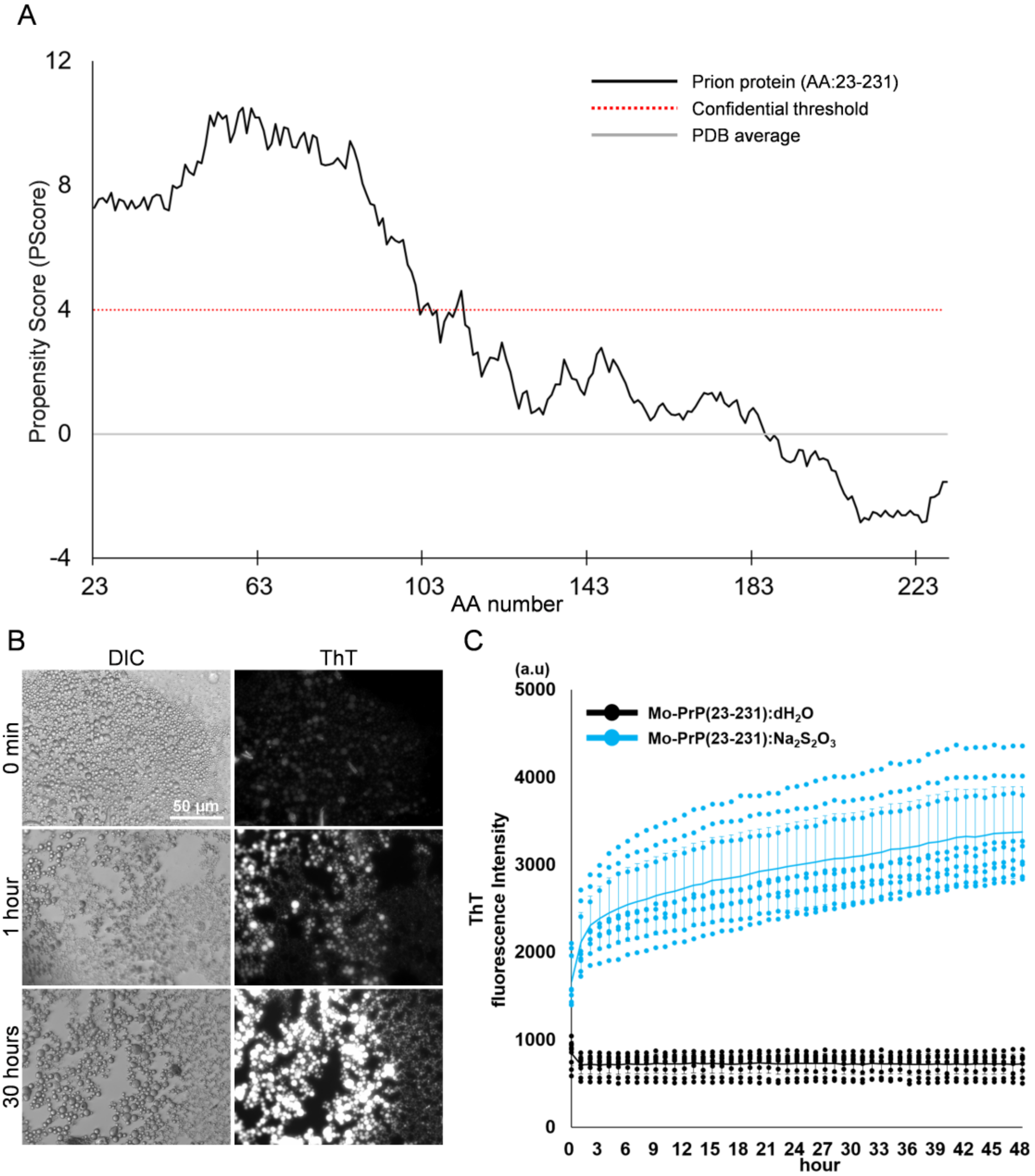


**Sup. Fig. 4 Mouse prion protein behaves similar to human prion protein.**

(A) Phase separation propensity score (PScore) based on planar π–π interactions. The black solid line indicates the PScore of human prion protein (residues 23–231) in each amino acid residue. The red dotted line indicates the confidence threshold. The gray solid line indicates the average score from the PDB average. The algorithm and dataset for the calculations are referred to as described previously (25).

(B) DIC and fluorescence microscopic images of Mo-rPrP (23–231): $dH_2O$ and $Na_2S_2O_3$ at 0 min, 1 h, and 30 h.

(C) ThT fluorescence intensity of Mo-rPrP (23–231): $dH_2O$ and Mo-rPrP (23–231): $Na_2S_2O_3$ measured after 48 h. Each dot represents a value measured in 3 independent experiments. Line indicates the average of each group. Error bars represent standard deviation. N=9.

**S-4**

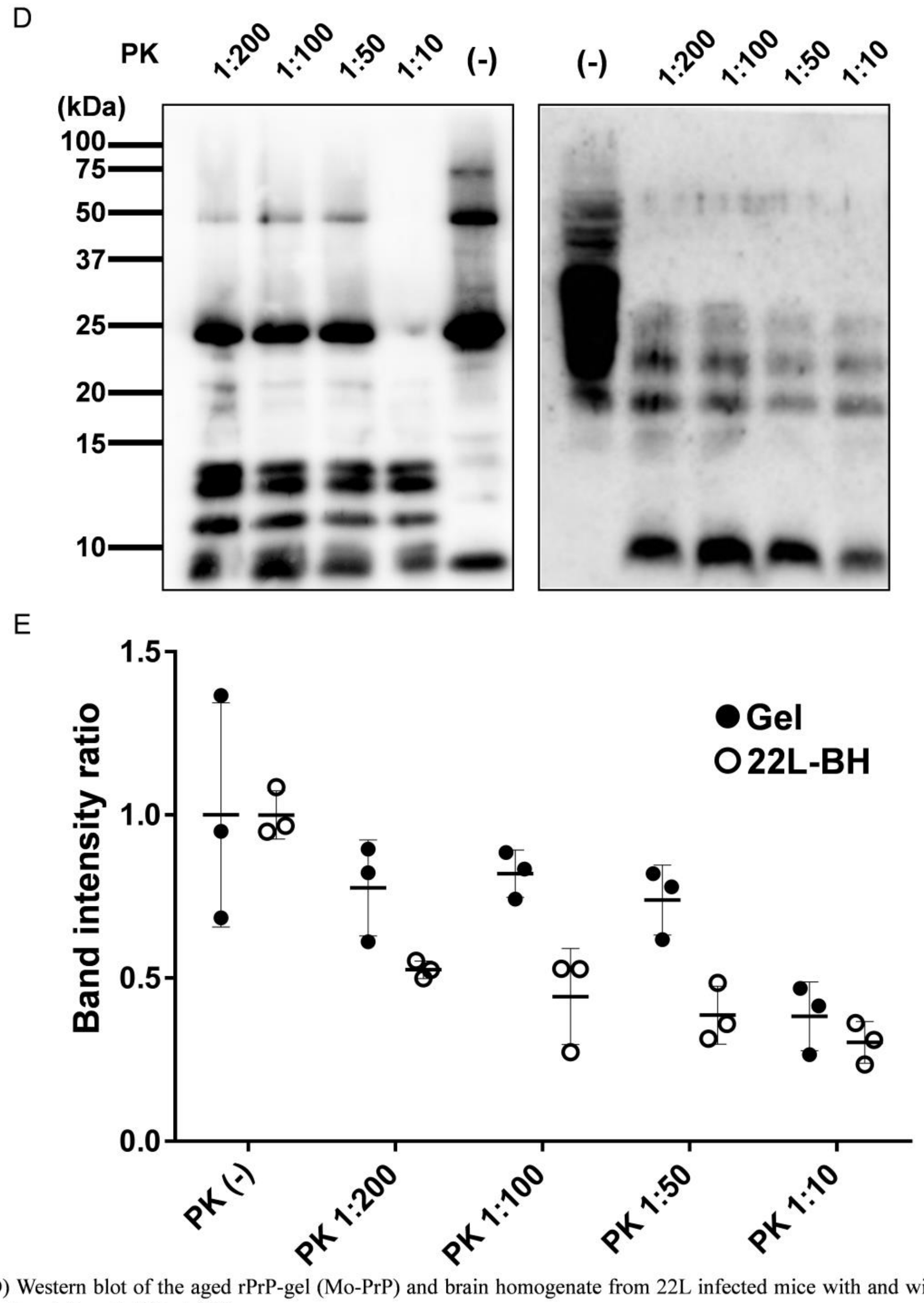


(D) Western blot of the aged rPrP-gel (Mo-PrP) and brain homogenate from 22L infected mice with and without PK treatment from 1:10 to 1:200.

(E) Quantification of band intensity ratio of the aged rPrP-gel (black circle) and brain homogenate from 22L infected mice (white circle). The average band intensity from the samples without PK treatment [PK (-)] was set to 1.0. The thin line shows SD, and the bold line shows the average. N=3, Bar: SD.

(F) Comparison of RT-QuIC detection of sCJD KinM brain homogenate, normal brain homogenate (NBH), and aged rPrP-gel(mo-rPrP). Each solid line corresponds to well (N=4).

**S-4**

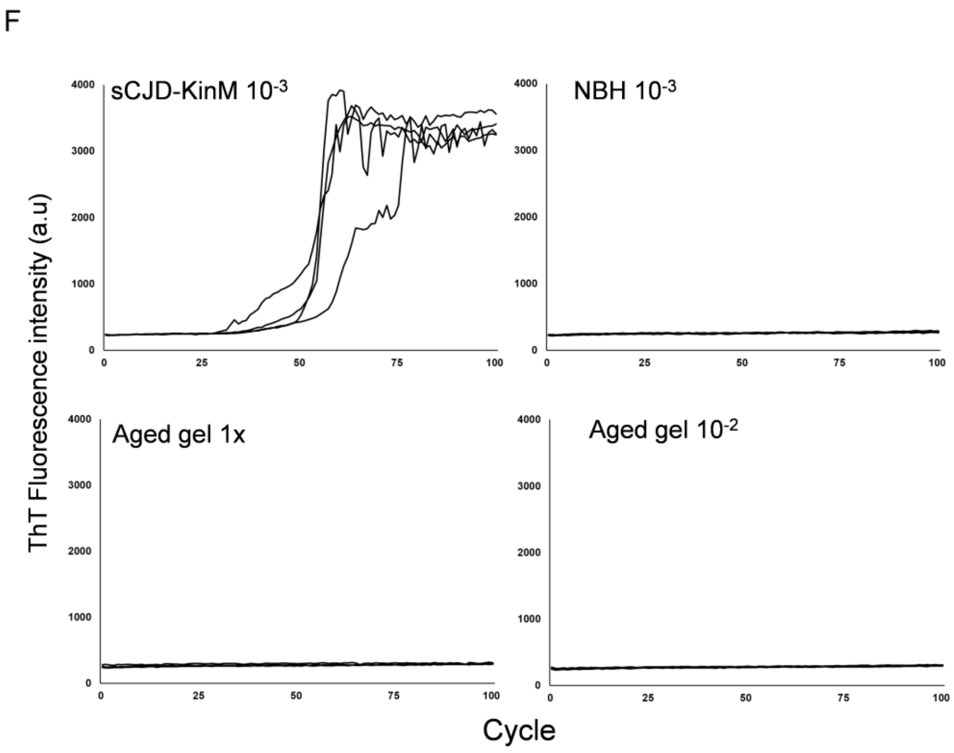


(F) Comparison of RT-QuIC detection of sCJD KinM brain homogenate, normal brain homogenate (NBH), and aged rPrP-gel(mo-rPrP). Each solid line corresponds to well (N=4).
